# Trust Me, I’m a Doctor: Examining Changes in How Privacy Concerns Affect Patient Withholding Behavior

**DOI:** 10.2196/jmir.6296

**Published:** 2017-01-04

**Authors:** Daniel M Walker, Tyler Johnson, Eric W Ford, Timothy R Huerta

**Affiliations:** ^1^ Department of Family Medicine The Ohio State University, College of Medicine Columbus, OH United States; ^2^ Department of Health Policy and Management Johns Hopkins Bloomberg School of Public Health Baltimore, MD United States

**Keywords:** privacy, electronic health records, disclosure, trust, electronic medical records, personal health information

## Abstract

**Background:**

As electronic health records (EHRs) become ubiquitous in the health care industry, privacy breaches are increasing and being made public. These breaches may make consumers wary of the technology, undermining its potential to improve care coordination and research.

**Objective:**

Given the developing concerns around privacy of personal health information stored in digital format, it is important for providers to understand how views on privacy and security may be associated with patient disclosure of health information. This study aimed to understand how privacy concerns may be shifting patient behavior.

**Methods:**

Using a pooled cross-section of data from the 2011 and 2014 cycles of the Health Information and National Trends Survey (HINTS), we tested whether privacy and security concerns, as well as quality perceptions, are associated with the likelihood of withholding personal health information from a provider. A fully interacted multivariate model was used to compare associations between the 2 years, and interaction terms were used to evaluate trends in the factors that are associated with withholding behavior.

**Results:**

No difference was found regarding the effect of privacy and security concerns on withholding behavior between 2011 and 2014. Similarly, whereas perceived high quality of care was found to reduce the likelihood of withholding information from a provider in both 2011 (odds ratio [OR] 0.73, 95% confidence interval [CI] 0.56-0.94) and 2014 (OR 0.61, 95% CI 0.48-0.76), no difference was observed between years.

**Conclusions:**

These findings suggest that consumers’ beliefs about EHR privacy and security, the relationship between technology use and quality, and intentions to share information with their health care provider have not changed. These findings are counter to the ongoing discussions about the implications of security failures in other domains. Our results suggest that providers could ameliorate privacy and security by focusing on the care quality benefits EHRs provide.

## Introduction

Electronic health records (EHRs) are now an omnipresent feature throughout the health care system, having been adopted by the majority of hospitals and physicians [1-4]. Moreover, new technologies such as health information exchange enable sharing of health records with other health care entities, and personal health records (PHRs) enable patient access to their health records [3]. As a result of this digitization, patients are more likely than ever to have their personal health information (PHI)—demographic information, medical history, and test and laboratory results—stored in an electronic format. In addition, a great deal of financial and other demographic data are collected and stored in a digital format for reimbursement purposes.

The growth in health information technology (HIT) use throughout the health care industry has aimed to improve care quality, as well as the efficiency of the health care system [5,6]. Health information technology can provide clinicians with more complete patient records at the point of care, enabling better clinical decision making, facilitating improved care coordination, and insuring patient safety as people move throughout the health care system [7]. HIT can also serve as a tool to enable better patient-provider communication, for example through secure messaging, leading to more patient-centered care [8,9]. Despite these potential benefits, recent high-profile, EHR security breaches reported in the media [10,11] make patients wary of this shift to the digital format [12,13]. Patients are concerned about the privacy of their information and its security as it is stored and transferred across the health care system [14-16]. These concerns can manifest themselves in a range of behaviors that can undermine the potential of the technology to facilitate improved care. In particular, Agaku et al found that patients deliberately withheld PHI from their provider due to concerns over the security of their EHR systems [17]. However, it is possible that quality perceptions can mediate this relationship. Campos-Castillo et al found that patients reporting higher quality of care experiences had a lower likelihood of withholding PHI out of privacy and security concerns [18]. However, due to limitations of the specific iteration of the dataset used by both authors, the study by Campos-Castillo et al did not include privacy and security concern items, and the study by Agaku et al did not include quality perceptions. Thus, while these 2 studies provide a foundation for understanding how patient concerns can manifest themselves in adverse behaviors, they examined different factors that taken together might result in different PHI withholding behaviors.

The purpose of this study was to build on the aforementioned 2 studies and advance the understanding of the factors that contribute to PHI withholding behavior. Moreover, this study examined changes in the influence of privacy and security concerns on PHI withholding behavior between 2 time points. As more new technologies facilitate data sharing across the health care system, it is essential to understand the factors that lead to patient mistrust of the health care system and to observe changes in this dynamic over time. Looking at an expanded set of factors that contribute to PHI withholding behaviors can help practitioners understand the relative strength of these factors in consumers’ minds. Such information can help providers and health care professionals respond to and mitigate patient privacy and security concerns in a manner that preserves the trust necessary to allow for high-fidelity PHI disclosure.

## Methods

### Sample

We created a pooled cross-section using data from both the 2011 and 2014 Health Information National Trends Survey (HINTS). The survey is administered as repeat cross-sections by the National Cancer Institute to a national sample of noninstitutionalized adults and gathers information regarding attitudes and perceptions about health information access and use [19]. HINTS maintains a core set of questions asked in each wave of the survey, but specific topic modules are included in separate cycles. Such is the case at-hand. The questions of interest were included only in the 2011 and 2014 surveys, restricting our research to these 2 cross-sections.

Both years of the survey were mail based. HINTS employed a stratified probability sample of the US adult, civilian, noninstitutionalized population. Addresses were randomly selected from the US Postal Service’s list of residential addresses, and then an adult within a selected household was chosen to respond to the survey using the next birthday method. The next birthday method asked for the adult in the household who would next have a birthday to complete the survey and was used to eliminate bias associated with the household member most likely to receive mail. A prepaid incentive was sent at the first mailing, and multiple follow-ups were sent to recipients in order to maximize the response rate. For household with a Hispanic last name, a Spanish version of the questionnaire was delivered in addition to the English version. The total number of respondents in the 2011 and 2014 surveys were 3959 and 3677, respectively. Taking into account survey design and weighting issues, the HINTS response rates were 36.67% (3959/10796) in 2011 and 34.44% (3677/10676) in 2014. Both survey iterations yielded samples that allowed for population-level inferences after adjustment. All respondents with complete responses for all variables of interest were included in the analytic sample.

### Measures

For the dependent variable, the HINTS survey asked whether the respondent had “ever kept information from (their) health care provider because (they) were concerned about the privacy and security of (their) medical record” (yes, no). This variable was used as the outcome variable for all analyses.

For our independent variables of interest, we closely followed the variable construction of related research [17,18]. Using data from the 2011 HINTS, Agaku et al evaluated the relationship between 4 indicators of privacy and security concerns and withholding behavior [17]. These 4 questions were also asked in the 2014 HINTS, making comparability possible between years. The 4 related questions about privacy and security concerns were as follows: do respondents have concerns about unauthorized access to their medical information when it is transferred electronically between providers; do respondents have concerns about unauthorized access to their medical information when it is faxed between health care providers; do they feel confident that safeguards are in place to protect their medical information from unauthorized access; and do they feel confident that they had a say in the collection, use, and sharing of their medical information. Respondents could answer each of these 4 questions in 3 levels: not at all concerned or confident, somewhat concerned or confident, or very concerned or confident. We tested differences in our model using all 3 levels and compared the results to using only 2 levels (not at all vs at least somewhat) and found no differences. Thus, for the purposes of simplicity, we dichotomized these variables.

Using the 2011 and 2012 HINTS surveys, Campos-Castillo et al identified a suppressor relationship between the perception that a provider had an EHR (“As far as you know, do any of your doctors or other health care providers maintain your medical information in a computerized system?”) and perceived global quality of care rating on withholding behavior [18]. This suppressor relationship occurred when 1 variable, the suppressor (eg, global quality of care rating), had a positive association with a covariate of interest (eg, perception of EHR use), but a negative relationship with the outcome variable (eg, withholding behavior). Accounting for the suppressor could reveal associations between the covariate and the outcome that might not have been detectable without controlling for the suppressor. Thus, we also included the perception that a provider had an EHR (yes, no) and perceived global quality of care rating as key independent variables of interest. The HINTS asked respondents who had a nonemergency department visit in the last 12 months to rate their perception of the quality of care they received using a Likert scale (poor, fair, good, very good, excellent). For comparability to the study by Campos-Castillo et al, the quality of care variable was left as a continuous variable and coded so that higher values indicated better care.

For comparability to the study by Campos-Castillo et al, our control variables aligned with their model [18]. The control variables captured respondents’ sociodemographic characteristics, health status, and health care utilization and preferences. Sociodemographic characteristics included race or ethnicity (white, black, Latino, other), gender (male, female), categorical age in years (18-35, 35-49, 50-64, 65-74, 75 or older), education level (less than high school, high-school, some college, college, graduate), annual category of household income (<US $20,000, US $20,000-$34,999, US $35,000-$49,999, US $50,000-$74,999, >US $75,000), an indicator for living in a rural area (defined as a nonmetro county), home-ownership status (homeowner, not homeowner), marital status (married, not married), any health insurance coverage (yes, no), immigration status (born in United States, immigrant), and employment status (employed, not employed). Items about patient health status included a self-rated general health measure (poor, fair, good, very good, excellent), an anxiety and depression index (none, mild, moderate, severe), and self-care self-efficacy (not confident, a little confident, somewhat confident, very confident, completely confident). Health care utilization and preferences included the number of nonemergency room visits in the year prior to the survey (1, 2-4, 5-9, 10 or more), regular health care provider (yes, no), perceived importance of personal health record access (not at all important, somewhat important, very important), and perceived importance that providers share data electronically (not at all important, somewhat important, very important).

### Analyses

Weighted, but unadjusted *t* tests or chi-squares were used to compare sample characteristics across the 2 years. Our analytic approach was designed to test the association of the independent variables of interest with withholding behavior in each year independently, as well as to test whether the relationship of the variables on withholding behavior changed between 2011 and 2014. To accomplish these tests, the 2 cross-sections were pooled together and a single fully interacted multivariate logit model, with each independent variable interacting with year, was estimated. This interacted model was solved to determine the adjusted odds ratios (OR) and confidence intervals (CI) within each year. The significance of the interaction term was used to evaluate the relative differences between years for each parameter. All results were weighted to yield US population-level inferences using a standard weighting approach developed for the HINTS dataset [20]. All analyses were conducted using Stata 14 (StataCorp LP) [21].

## Results

Overall, 2217 respondents from 2011 had complete information and were included in the analytic sample, and 2176 respondents from 2014 were included. Demographic characteristics in each year are displayed in Table 1, along with the results from a chi-square test to show any differences between years.

The dependent variable of interest for this study was whether the respondent had ever withheld any PHI from a medical provider out of privacy or security concerns. No difference in the level of this behavior was observed between years: in 2011, 14.79% (328/2217) of respondents reported this behavior, whereas in 2014, 14.93% (325/2176) of respondents reported withholding information from their provider out of privacy concerns (Table 2). Comparison of the rates of additional variables of interest between 2011 and 2014, including attitudes concerning privacy and security, quality perceptions, and health care utilization, are presented in Table 2.

To test the hypothesis that the relationship between the withholding behavior and the attitudinal variables and quality perceptions was unchanged between years, the 2 cross-sections of data were pooled and a fully interacted multivariate model predicting withholding behavior was estimated and solved for each different level of year. The interaction terms allowed for testing the relative differences of each parameter between the 2 years (see Table 3 for adjusted ORs; see Multimedia Appendix 1 for average marginal effects). This analysis revealed no changes between 2011 and 2014 in the association of privacy and security attitudes on withholding behavior. No effect of concerns regarding unauthorized access to electronic medical information on withholding behavior in either year was observed, and no difference in this effect between years was found. While concerns about unauthorized access to faxed medical information on withholding behavior was found to be significant in both 2011 and 2014, no difference in this effect was found between years. Respondent confidence that safeguards were in place to protect their medical information was not related to withholding behavior in either year, and again no difference was found between years. Lastly, there was no effect on respondent confidence that they had some control over their medical information on withholding behavior in either year, and no difference was found between the 2 years.

The perception of greater quality of care was found to significantly lower the odds of withholding behavior in both 2011 and 2014, but no difference was observed between years. Provider having an EHR was not found to be related to withholding behavior in either 2011 or 2014, and no difference was observed between years.

**Table 1:** Demographic characteristics of the analytic sample in 2011 and 2014. Frequencies and test statistics were adjusted for survey weights.

Variable		2011 (n=2217), n (%)	2014 (n=2176), n (%)	*P* value
**Sex**				.30
	Female	1550 (53.94)	1407 (55.48)	
	Male	1024 (46.06)	834 (44.52)	
**Race**				.57
	White	1736 (69.76)	1403 (71.51)	
	Black	370 (10.28)	355 (10.02)	
	Latino	272 (12.93)	321 (12.51)	
	Other	176 (7.02)	154 (5.96)	
**Education**				<.001
	Less than high school	191 (10.48)	159 (9.14)	
	High school	455 (18.79)	353 (15.63)	
	Some college	796 (33.88)	698 (31.11)	
	College	666 (21.61)	613 (26.49)	
	Graduate	466 (15.24)	418 (17.62)	
**Age (years)**				.59
	18-35	408 (30.70)	309 (29.52)	
	35-49	629 (27.05)	500 (28.55)	
	50-64	912 (26.55)	810 (25.62)	
	65-74	379 (9.09)	388 (9.48)	
	75+	246 (6.63)	235 (6.88)	
Employed		1380 (58.25)	1175 (62.16)	.08
**Income**				<.001
	<US $20,000	482 (19.71)	441 (16.58)	
	US $20,000-$34,999	416 (16.37)	290 (10.35)	
	US $35,000- $49,999	378 (13.07)	339 (15.08)	
	US $50,000-$74,999	453 (17.46)	405 (18.20)	
	>US $75,000	845 (33.40)	766 (39.79)	
Married		1436 (53.93)	1114 (54.97)	.49
Rural		409 (16.33)	300 (16.54)	.91
US Immigrant		318 (11.90)	298 (11.60)	.82
Homeowner		1805 (61.35)	15.2 (62.59)	.82
Health insurance		2415 (87.92)	2039 (90.80)	.04

**Table 2:** Withholding behavior, privacy and security concerns, health and quality perceptions, and health care utilization compared between 2011 and 2014. Frequencies and test statistics were adjusted for survey weights.

Variable		2011 (n=2217), n (%)	2014 (n=2176), n (%)	*P* value
Withheld information		328 (14.80)	325 (14.90)	.22
**Electronic information safe**				.17
	Not at all	824 (37.95)	711 (34.64)	
	At least somewhat concerned	1472 (62.05)	1529 (65.36)	
**Faxed information safe**				.07
	Not at all	781 (34.39)	633 (30.47)	
	At least somewhat concerned	1538 (65.61)	1601 (69.53)	
**Confident safeguards exist**				.01
	Not at all confident	560 (25.85)	487 (19.72)	
	At least somewhat confident	1758 (74.15)	1747 (80.28)	
**Control over use of information**				.37
	Not at all confident	677 (29.45)	623 (27.38)	
	At least somewhat confident	1644 (70.55)	1614 (72.62)	
Quality of care (mean+SE)		4.01 (0.03)	4.03 (0.04)	.87
**Important that providers share electronic health record data**				.71
	Not at all	128 (4.94)	102 (5.83)	
	Somewhat	704 (29.25)	615 (28.22)	
	Very	1742 (65.81)	1524 (65.94)	
**Important to have access to personal health record**				.18
	Not at all	192 (7.96)	147 (5.80)	
	Somewhat	589 (21.62)	512 (22.05)	
	Very	1793 (70.42)	1582 (72.15)	
Perceived provider electronic health record use		2303 (88.31)	2134 (94.54)	<.001
**General health**				.14
	Poor	79 (2.39)	85 (2.10)	
	Fair	315 (11.96)	297 (10.16)	
	Good	909 (33.85)	845 (39.12)	
	Very good	959 (38.09)	773 (34.57)	
	Excellent	312 (13.71)	241 (14.05)	
**Depression**				.24
	None	1725 (66.84)	1584 (71.37)	
	Mild	503 (19.67)	404 (17.86)	
	Moderate	197 (7.15)	144 (6.11)	
	Severe	149 (6.34)	109 (4.65)	
**Nonemergency room visits in past year**				.18
	1	451 (21.54)	375 (19.30)	
	2-4	1398 (53.25)	1269 (58.40)	
	5-9	460 (15.87)	362 (13.13)	
	≥10	265 (9.34)	235 (9.17)	
Have a regular provider		2054 (73.42)	1736 (73.24)	.93
Self-care efficacy, mean (SE)		3.87 (0.03)	3.85 (0.03)	.80

**Table 3:** Comparison of patient attitudes and demographic variables that are associated with withholding behavior in 2011 and 2014 based on a fully interacted model with a pooled cross-section (N=4393; model adjusted for survey weights).

Variable		2011 Odds ratio (95% CI)	2014 Odds ratio (95% CI)	Significance of interaction
**Electronic information safe**	
	Not at all	Ref^a^	Ref	
	At least somewhat concerned	1.63 (0.74-3.62)	1.82 (0.78-4.22)	.85
**Faxed information safe**	
	Not at all	Ref	Ref	
	At least somewhat concerned	7.09 (2.56-19.66)^b^	3.27 (1.37-7.83)^c^	.25
**Confident information safe**	
	Not at all confident	Ref	Ref	
	At least somewhat confident	0.73 (0.34-1.57)	1.54 (0.66-3.60)	.20
**Control information**				
	Not at all confident	Ref	Ref	
	At least somewhat confident	1.71 (0.91-3.21)	1.10 (0.55-2.20)	.35
Quality of care		0.72 (0.56-0.94)^c^	0.61 (0.48-0.76)^b^	.30
**Important that providers share electronic health record data**				
	Not at all	Ref	Ref	
	Somewhat	0.77 (0.26-2.34)	0.56 (0.10-3.02)	.74
	Very	0.58 (0.21-1.62)	0.72 (0.14-3.61)	.83
**Important that you have access to personal health record**				
	Not at all	Ref	Ref	
	Somewhat	0.31 (0.08-1.19)	1.31 (0.26-6.52)	.17
	Very	0.47 (0.13-1.69)	1.81 (0.45-7.30)	.16
**Provider has an electronic health record**		1.47 (0.79-2.74)	0.70 (0.30-1.66)	.17
**Sex**				
	Female	Ref	Ref	
	Male	0.84 (0.58-1.24)	1.10 (0.67-1.80)	.40
**Race**				
	White	Ref	Ref	
	Black	1.63 (0.75-3.55)	0.98 (0.43-2.24)	.37
	Latino	1.21 (0.53-2.78)	1.37 (0.53-3.57)	.84
	Other	2.28 (0.89-5.84)	1.97 (0.81-4.81)	.82
**Education**				
	Less than high school	Ref	Ref	
	High school	0.56 (0.19-1.68)	1.16 (0.40-3.36)	.35
	Some college	1.14 (0.40-3.24)	0.90 (0.37-2.19)	.73
	College	0.80 (0.27-2.40)	0.86 (0.33-2.24)	.92
	Graduate	1.18 (0.38-3.69)	1.43 (0.57-3.63)	.79
**Age (years)**				
	18-35	Ref	Ref	
	35-49	1.60 (0.83-3.09)	1.03 (0.49-2.20)	.39
	50-64	1.11 (0.54-2.26)	0.63 (0.31-1.30)	.27
	65-74	0.87 (0.38-1.98)	0.59 (0.24-1.48)	.54
	75+	0.67 (0.18-2.50)	0.26 (0.07-0.94)^c^	.32
Employed		1.79 (0.96-3.35)	1.53 (0.83-2.84)	.83
**Income**				
	<US $20,000	Ref	Ref	
	US $20,000- $ 34,999	0.76 (0.30-1.93)	0.97 (0.43-2.21)	.70
	US $35,000- $49,999	0.57 (0.23-1.42)	0.99 (0.39-2.54)	.40
	US $50,000- $74,999	0.77 (0.35-1.69)	0.84 (0.38-1.85)	.87
	>US $75,000	0.55 (0.24-1.25)	0.68 (0.29-1.60)	.72
Married		0.79 (0.50-1.24)	0.72 (0.41-1.26)	.85
Rural		1.00 (0.54-1.86)	1.18 (0.47-2.92)	.77
US immigrant		1.01 (0.55-1.88)	0.73 (0.37-1.41)	.46
Homeowner		1.21 (0.67-2.21)	0.71 (0.41-1.23)	.19
Health insurance		1.35 (0.47-3.88)	0.95 (0.41-2.21)	.61
**General health**				
	Poor	Ref	Ref	
	Fair	1.26 (0.27-5.92)	0.41 (0.12-1.39)	.25
	Good	1.50 (0.33-6.72)	0.28 (0.08-1.03)	.10
	Very good	2.13 (0.46-9.77)	0.41 (0.11-1.50)	.11
	Excellent	2.16 (0.44-10.67)	0.49 (0.10-2.31)	.19
**Depression**				
	None	Ref	Ref	
	Mild	1.14 (0.61-2.13)	0.87 (0.42-1.81)	.57
	Moderate	2.71 (1.14-6.42)^c^	1.85 (0.59-5.79)	.59
	Severe	1.13 (0.39-3.26)	1.13 (0.42-3.01)	.99
**Nonemergency room visits in past year**				
	1	Ref	Ref	
	2-4	0.91 (0.52-1.60)	1.06 (0.49-2.29)	.75
	5-9	1.08 (0.50-2.32)	1.02 (0.39-2.65)	.92
	≥10	1.10 (0.45-2.73)	1.66 (0.66-4.18)	.53
Have a regular provider		0.98 (0.56-1.72)	1.75 (0.95-3.23)	.17
Mean self-care efficacy		0.97 (0.69-1.38)	0.97 (0.65-1.44)	.97

^a^Ref: reference category.

^b^*P*<.001.

^c^*P*<.05.

## Discussion

### Principal Findings

Public perception of the safety of their medical records is critical to not only encouraging full disclosure to their health care provider, but also supporting adoption and use of electronic modes of health communication made available by new technologies. Distrust can lead to withholding of information from providers and undermine the delivery of high-quality, efficient care. The aim of our analysis was to determine the factors that contribute to this withholding behavior, and how the effect of these factors may be shifting over time. In short, we found that the association between patient concerns and withholding information from a provider remained unchanged between 2011 and 2014.

Earlier work using 2011 HINTS data found that respondents with concerns about both faxed and electronic data, and lack of confidence that safeguards were in place to protect medical information, were more likely to withhold information from their provider [17]. Our model included a more extensive set of control variables, as well as indicators of quality and provider EHR use, and found no such relationships to exist in either 2011 or 2014. This discordance between the 2 analyses suggests that patient concerns over the safety of their medical information may not be adversely related to their disclosure of PHI to their providers. Alternatively, other factors beyond general privacy and security concerns may lead to withholding behaviors, such as lack of trust, stigma, or concerns about insurance rates [22-24].

In contrast to the lack of replicability of the earlier findings regarding the relationship between security and privacy concerns, and withholding behavior, our analysis did observe findings similar to those of Campos-Castillo et al regarding the association between quality and withholding behavior [18]. The original work did not include the privacy and security questions used in this analysis, but did include an identical set of covariates. Nonetheless, Campos-Castillo et al found that perceptions of greater quality of care reduced the odds of PHI withholding behavior [18]. This relationship was observed in our study in both 2011 and 2014, effectively reinforcing the earlier work of Campos-Castillo et al [18]. Interestingly, no difference was observed in the correlation between quality and withholding behavior between years. This latter finding suggests that despite the rise in ubiquity of EHRs alongside more public privacy breaches, high perceived quality of care may still trump any concerns that contribute to withholding behavior.

Related to this issue, our study did observe an increase in perceived EHR use between 2011 and 2014. Despite similar findings regarding quality perceptions between our study and the study by Campos-Castillo et al, our work found no relationship between perceived provider EHR use and withholding behavior, while their study did find the presence of such relationship [18]. To be specific, the study by Campos-Castillo et al found that quality acted as a suppressor variable that moderated the relationship between perceived EHR use and patient withholding behavior [18]. The variables regarding attitudes about EHR and privacy and security may be acting as confounders in the relationship, which would explain the significance in the earlier study but not this one.

Overall, our analysis suggests that in spite of the existence of security and privacy concerns, focusing resources on the delivery of high-quality care may be an effective strategy to foster patient trust. Patients may perceive quality as an indicator of a provider’s carefulness with their medical information. Quality may also help to build the patient-provider relationship [25,26]. Alternatively, the notion of privacy is evolving as more and more personal information is held on the Internet [27,28]. It may be possible that given the increasing digitization of personal information, the US population is willing to accept greater amounts of privacy risks of their personal data as a trade-off for greater convenience or better quality of care. This is a ripe area for future research, as the field of health services research should consider the role that changes in collective ideas of privacy may be playing in how patients relate to the health care system.

### Limitations

Our analysis faced 3 important limitations. First, the administration of HINTS combined with the weighting technique allowed for the survey to be nationally representative. However, selection bias might have remained that limited the generalizability of the sample. Furthermore, our dependent variable (withholding behavior) could be related to survey response, and respondents with complete answers to all questions might systematically differ from nonrespondents. Related to this issue, because the HINTS survey was administered to only noninstitutionalized individuals, the findings regarding withholding behavior might be biased toward the outpatient environment. Continued monitoring of the factors that contribute to patient withholding with future iterations of HINTS can help to assess the impact of this bias and evaluate the true relationship between the variables of interest and the outcome.

Second, all information in HINTS was self-reported, potentially resulting in unreliable responses. This concern was particularly relevant to the withholding behavior question that was subject to social desirability bias [29]. Third, while our analysis did compare data from 2 different years, the cross-sectional nature of HINTS made the determination of causal inference challenging. Specifically, HINTS asked whether a person had *ever* withheld information from their provider, leaving open the possibility that withholding behavior preceded concerns about privacy and security, or quality perceptions. This issue was further complicated by the ordering of the questions in both cycles of the HINTS survey used in this study, where the question about perception of quality of care preceded the questions about perceived EHR use and withholding behavior. Furthermore, the frame of reference for the quality of care question was “…in the past 12 months,” while for EHR use or withholding behavior question, the frame of reference was undefined or “ever.” As a result, the responses to these questions might have drawn on different experiences, and might not necessarily reference the same encounter with the health care system. Thus, our findings regarding quality of care might be biased away from the null, and our study results should be interpreted with these measurement limitations in mind. However, despite the inability to detect causality, the national representativeness of HINTS makes it useful to identify macro trends at the individual variable level. Future studies that examine the effect of privacy and security concerns on patient withholding behavior may take a more micro approach, potentially using in-depth interviews to better understand how these concerns may manifest themselves and to identify specific omitted factors.

### Conclusions

Monitoring and assessing how technological advances may be related to patient behavior is critical to insure high-quality care and patient safety. In contrast to previous findings, the analysis presented in this study suggested minimal effects of privacy and security concerns on PHI withholding behavior, and that this relationship was constant over time. Similarly, the relationship between quality perceptions and withholding behavior was also constant over time, yet negatively correlated at both time points. Thus, our findings suggested that improving quality can buffer privacy and security concerns. While technological safeguards to protect patient health information remains important, health professionals should not forget that individual relationships remain the foundation of the patient’s experience with the health care system.

## Appendix

Multimedia Appendix 1 Average marginal effects of patient attitudes and demographic variables that are associated with withholding behavior, in 2011 and 2014, based on a fully interacted model with a pooled cross-section (n=4393).

## Abbreviations

EHR: electronic health record
HINTS: Health Information and National Trends Survey
HIT: health information technology
PHI: personal health information
PHR: personal health record
