# Supplementary material for: Trust Me, I’m a Doctor: Examining Changes in How Privacy Concerns Affect Patient Withholding Behavior
Source: J Med Internet Res. 2017 Jan 4;19(1):e2. doi: 10.2196/jmir.6296 (PMC5244032; doi:10.2196/jmir.6296)
Supplement: Multimedia Appendix 1 [file jmir_v19i1e2_app1.pdf]

**Multimedia Appendix 1:** Average marginal effects of patient attitudes and demographic variables that are associated with withholding behavior, at 2011 and 2014, based on a fully interacted model with a pooled cross-section (n=4,393).[a]

| Variable | 2011 OR (95% CI) | 2014 OR (95% CI) |
|---|---|---|
| **Electronic Info Safe** | | |
| Not at All | Ref. | Ref. |
| At least Somewhat Concerned | 4.21% | 5.60% |
| **Faxed Info Safe** | | |
| Not at All | Ref. | Ref. |
| At least Somewhat Concerned | 12.62%*** | 10.03%*** |
| **Confident Info Safe** | | |
| Not at All Confident | Ref. | Ref. |
| At least Somewhat Confident | -3.04% | 4.05% |
| **Control Info** | | |
| Not at All Confident | Ref. | Ref. |
| At least Somewhat Confident | 4.60% | 0.98% |
| **Quality of Care** | -3.00%** | -5.07%*** |
| **Important that Providers Share EMR Data** | | |
| Not at all | Ref. | Ref. |
| Somewhat | -2.81% | -6.21% |
| Very | -5.56% | -3.77% |
| **Important that you Have Access to PHR** | | |
| Not at All | Ref. | Ref. |
| Somewhat | -12.39% | 2.21% |
| Very | -8.71% | 5.39% |
| **Provider has an EMR** | 3.28% | -3.85% |
| **Sex** | | |
| Female | Ref. | Ref. |
| Male | -1.58% | 0.95% |
| **Race** | | |
| White | Ref. | Ref. |
| Black | 4.84% | -0.19% |
| Latino | 1.73% | 3.34% |
| Other | 8.98% | 7.84% |
| **Education** | | |
| Less than High school | Ref. | Ref. |
| High School | -4.76% | 1.52% |
| Some College | 1.35% | -0.99% |

| | | |
|---|---|---|
| College | -2.03% | -1.45% |
| Graduate | 1.69% | 3.99% |
| **Age** | | |
| 18-35 | Ref. | Ref. |
| 35-49 | 4.64% | 0.39% |
| 50-64 | 0.91% | -4.65% |
| 65-74 | -1.16% | -5.16% |
| 75+ | -3.06% | -10.51%* |
| **Employed** | 5.20%* | 4.19% |
| **Income** | | |
| <$20,000 | Ref. | Ref. |
| $20,000 – 34,999 | -2.88% | -0.31% |
| $35,000 – 49,999 | -5.50% | -0.06% |
| $50,000 – 74,999 | -2.77% | -1.83% |
| >$75,000 | -5.82% | -3.82% |
| **Married** | -2.23% | -3.34% |
| **Rural** | 0.02% | 1.68% |
| **US Immigrant** | 0.13% | -3.03% |
| **Homeowner** | 1.79% | -3.58% |
| **Health Insurance** | 2.59% | -0.56% |
| **General Health** | | |
| Poor | Ref. | Ref. |
| Fair | 1.67% | -11.80% |
| Good | 3.05% | -15.42% |
| Very Good | 6.38% | -11.70% |
| Excellent | 6.55% | -9.83% |
| **Depression** | | |
| None | Ref. | Ref. |
| Mild | 1.24% | -1.38% |
| Moderate | 11.50%* | 7.21% |
| Severe | 1.07% | 1.25% |
| **Non-Emergency Room Visits in Past Year** | | |
| 1 | Ref. | Ref. |
| 2-4 | -0.88% | 0.56% |
| 5-9 | 0.75% | 0.19% |
| ≥10 | 0.95% | 5.58% |
| **Have a Regular Provider** | -0.20% | 5.32% |
| **Mean Self Care Efficacy** | -0.24% | -0.36% |

**Notes:** ****P* < .001; ** *P* < .01; * *P* < .05; OR = Odds Ratio; CI = Confidence Interval.[a]Model adjusted for survey weights.
